# Supplementary material for: Cryo-electron tomography pipeline for plasma membranes
Source: Nat Commun. 2025 Jan 20;16:855. doi: 10.1038/s41467-025-56045-z (PMC11747107; doi:10.1038/s41467-025-56045-z)
Supplement: Supplementary file 1 — Supplementary Information [file 41467_2025_56045_MOESM1_ESM.pdf]

Supplementary Information

Cryo-electron tomography pipeline for plasma membranes.

Willy W. Sun[1, †], Dennis J. Michalak[1, †], Kem A. Sochacki[1, †*], Prasanthi Kunamaneni[1,2], Marco A. Alfonzo-Méndez[1], Andreas M. Arnold[1], Marie-Paule Strub[1], Jenny E. Hinshaw[2*], Justin W. Taraska[1*]

[1]National Heart, Lung, and Blood Institute, US National Institutes of Health, Bethesda, Maryland, USA

[2]National Institute of Diabetes and Digestive and Kidney Diseases, US National Institutes of Health, Bethesda, Maryland, USA

† These authors contributed equally

*These authors jointly supervised this work

kem.sochacki@nih.gov, jennyh@niddk.nih.gov, justin.taraska@nih.gov

**Supplementary Figures**	
Figure S1	Cell unroofing setup.
Figure S2	The unroofing process with step-by-step photos.
Figure S3	The pressure of the unroofing buffer is the primary parameter that affects cell unroofing.
Figure S4	Evaluating cells on EM grids and FIB milling of vitrified HSC3 cells.
Figure S5	Platinum replica electron microscopy of different HSC3 cell plasma membrane isolation preparations.
Figure S6	A large print of a 12.3 µm x 8 µm montage of an isolated basal plasma membrane from a HSC3 cell on an R2/1 Quantifoil grid.
Figure S7	A large print of a 12.3 µm x 8 µm montage of an isolated apical plasma membrane from a HSC3 cell on an R2/1 Quantifoil grid.
Figure S8	Tomogram thickness.
Figure S9	Visual representation of the subtomogram averaging workflow.
Figure S10	ER-bound polysome in unroofed membrane tomogram.
Figure S11	Western Blot Analysis of protein transfection.
**Supplementary Methods**	
**Supplementary References**	

Fig. S1

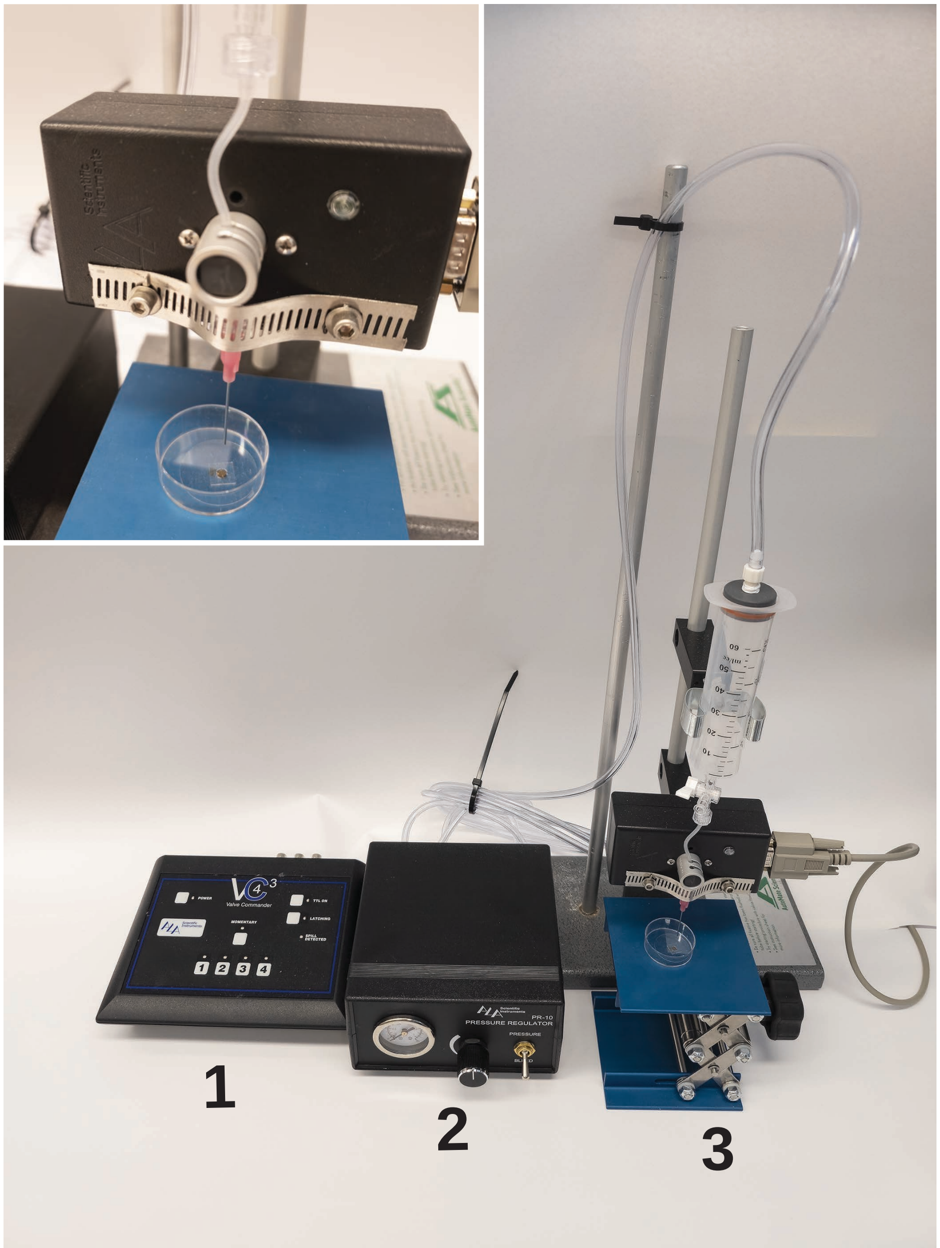

Figure S1. Cell unroofing setup. The unroofing system consists of valve control (1), pressure control (2), and a syringe (3) that applies a pressurized stream onto a cell-containing EM grid. The inset shows a close-up view of the EM grid-stencil-coverslip setup.

Figure S2.

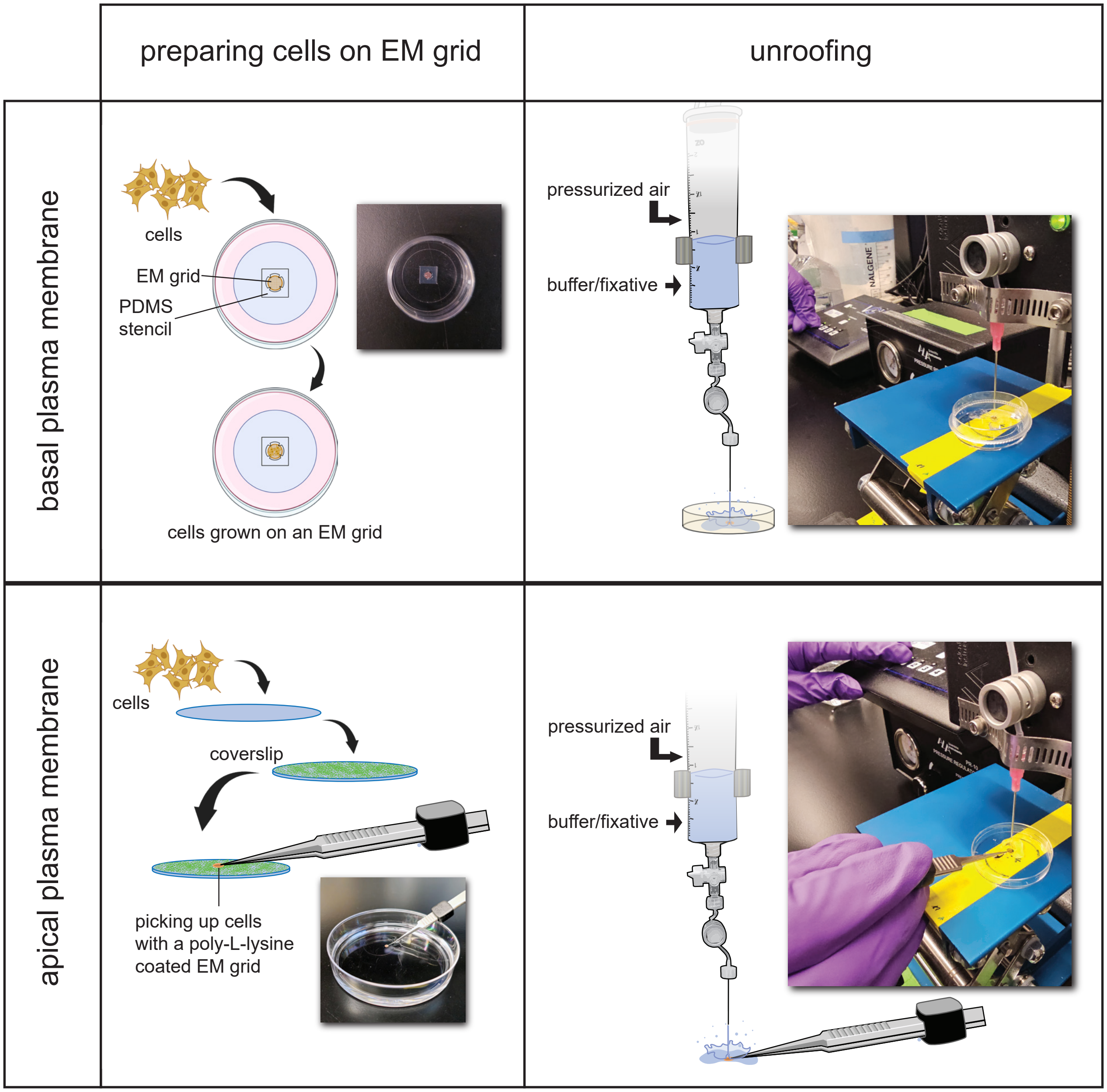

Figure S2. The sample preparation steps are accompanied by photographs for better clarity of the unroofing process. Created in part using icons from BioRender. Created in BioRender. Sun, W. (2025) https://BioRender.com/s94x049

Figure S3.

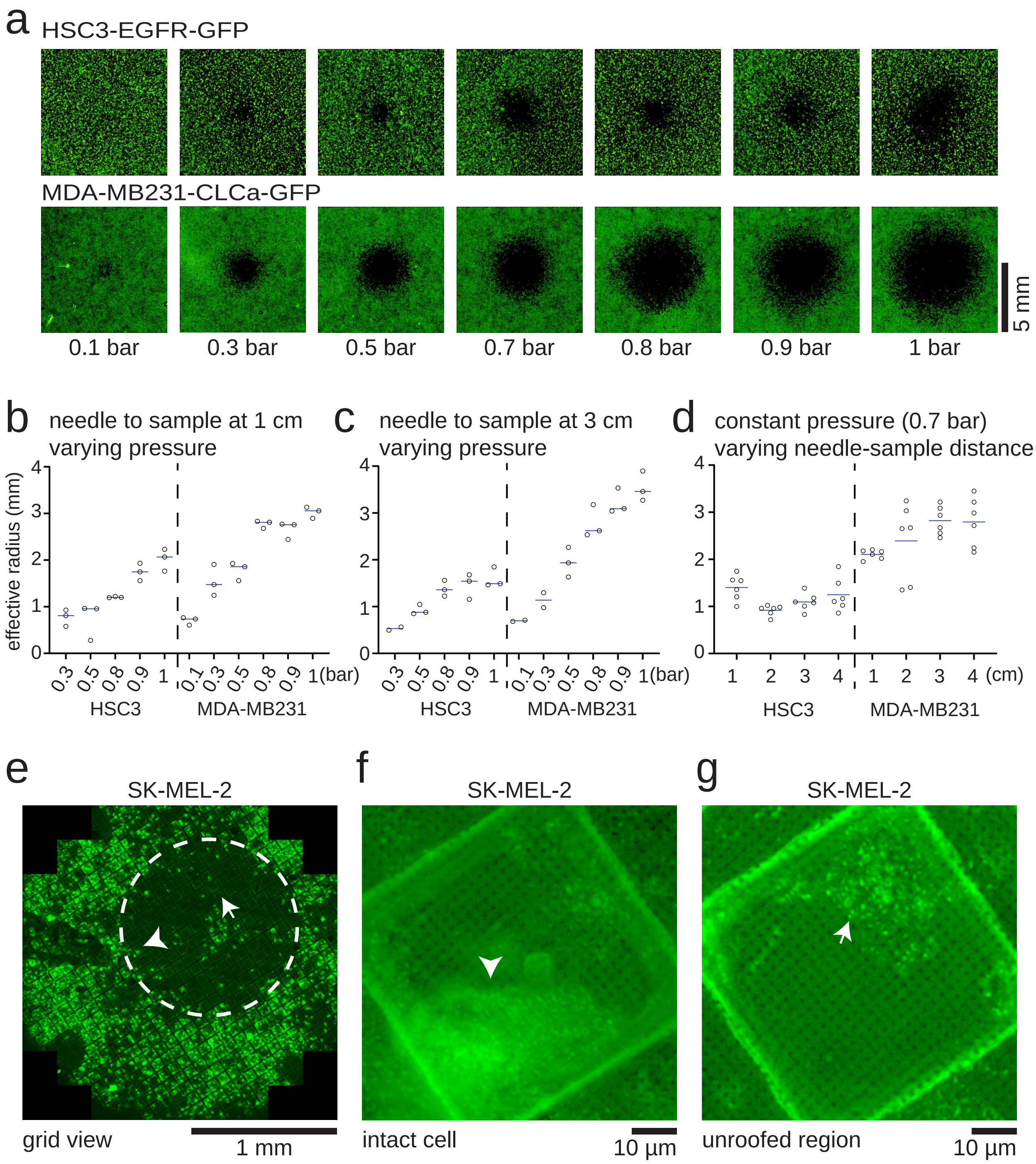

Figure S3. The pressure of the unroofing buffer is the primary parameter that affects cell unroofing. **a,** A panel of montages of HSC3-EGFR-GFP and MDA-MB231-CLCa-GFP cells unroofed at a fixed needle-to-sample distance (1 cm) at varying pressures from 0.1 bar to 1 bar. Increasing the pressure of the unroofing buffer increases the effective radius in both cell types. **b-d,** Testing how needle-to-sample distances and different pressures affect unroofing. **b,** A scatter plot showing the effect of increasing the pressure of the unroofing buffer while the needle-sample position is held at 1 cm. **c,** A graph showing how different pressure conditions affect unroofing with the needle-sample distance at 3 cm. **d,** A plot showing the effective radius of cell unroofing under the condition of constant pressure (0.7 bar) and varying needle-to-sample distance (from 1 cm to 4 cm in 1 cm increments). Blue horizontal bars depict the means. Six coverslips were unroofed at each needle-sample distance in **d**. Three coverslips were unroofed at each pressure setting in **b** and **c**. 0.1 bar does not appear to be a sufficient pressure to unroof HSC3 cells across six coverslips, three at 1 cm and three at 3 cm. One coverslip of HSC3 cells did not get unroofed at 0.3 bar at 3 cm. Two coverslips of MDA-MB231 cells did not undergo unroofing, one at 0.1 bar at 3 cm and one at 0.3 bar at 3 cm. **e,** A CLEM image showing an unroofed grid of SK-MEL-2-clca-GFP (clathrin light chain tagged with GFP) cells unroofed with a needle-sample distance of 1 cm and a pressure of 0.7 bar. The white dashed circle denotes the unroofed region. The white arrowhead shows a grid hole with an intact cell; an enlarged view is shown in **f**. The white arrow points to a grid hole with an isolated basal plasma membrane; an enlarged view is shown in **g**.

Figure S4.

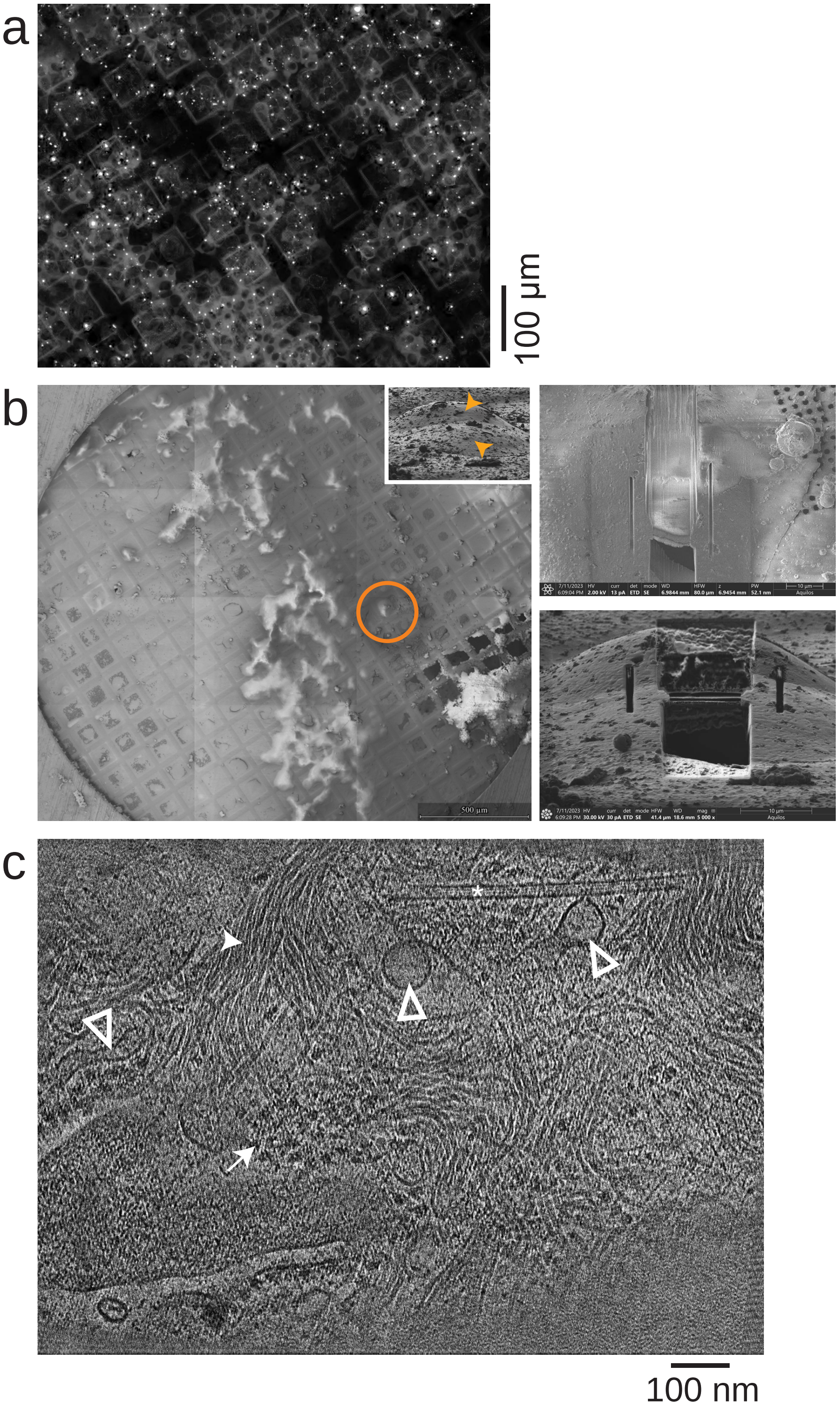

Figure S4. Evaluating cells on EM grids and FIB milling of vitrified HSC3 cells. a, A fluorescent image from Leica CLEM showing intact HEK293 cells transfected with Dynamin-K44A-GFP on a poly-L-lysine coated EM grid. **b,** A series of images showing the FIB-milling workflow. HSC3 cells were picked up by a poly-L-lysine coated EM grid and plunge frozen. In the left panel, backscattered electron view and ion view (inset) show the overview of a vitrified EM grid with picked-up HSC3 cells and a close-up view (orange circle) of two cells in a grid hole (orange arrows). The right panels show the backscattered electron (right-top) and ion (right-bottom) views of a finely milled lamella. **c,** A tomographic projection from a tomogram collected from a lamella. A variety of features are preserved in an HSC3 cell, including a microtubule (*), ribosomes (white arrow), intermediate filaments (white arrowhead), and membrane organelles (white empty arrowheads).

Figure S5.

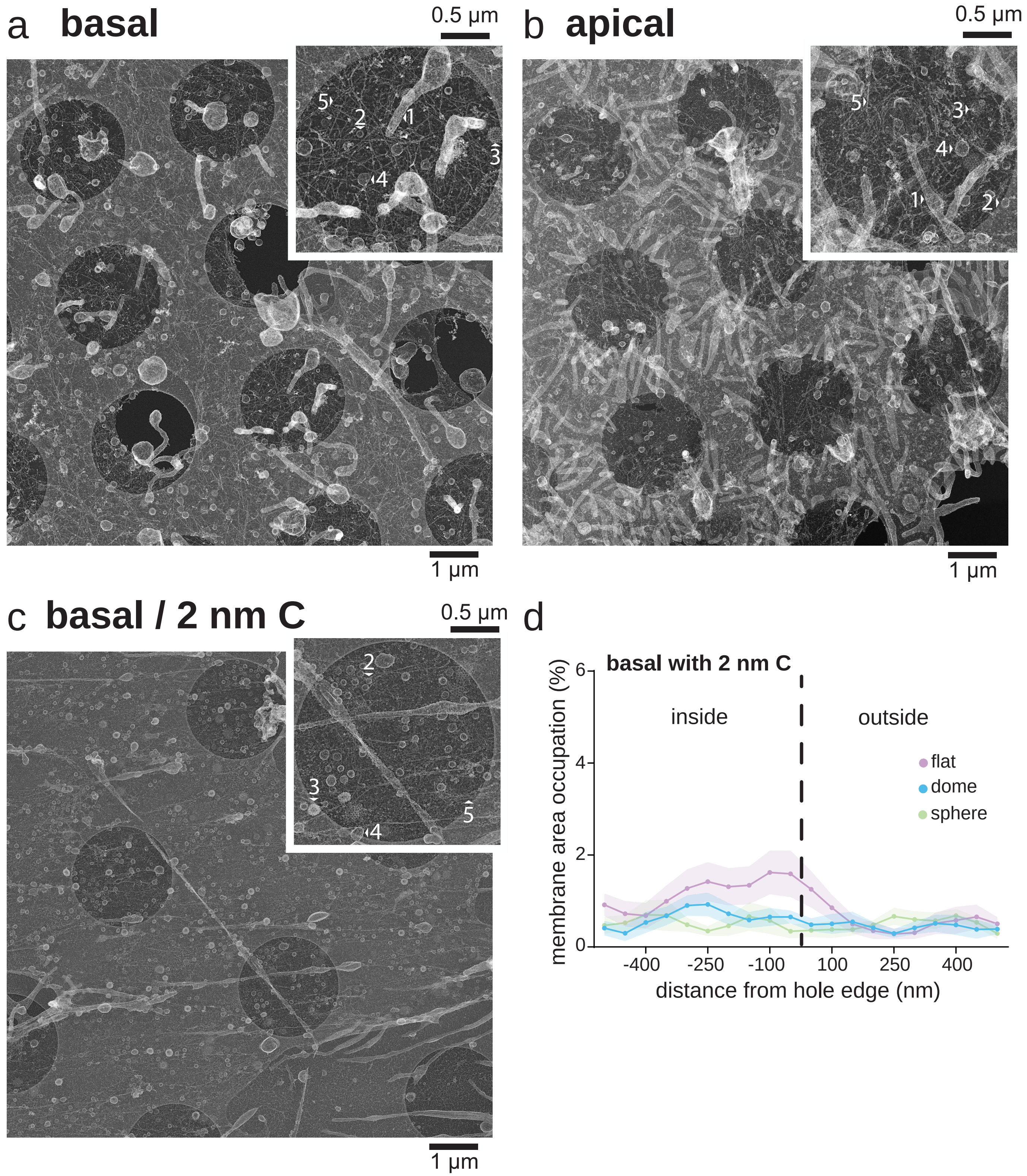

Figure S5. Platinum replica electron microscopy of different HSC3 cell plasma membrane isolation preparations. a, An HSC3 basal plasma membrane on a Quantifoil R2/1 Au grid with an enlarged crop-out. 1=filopodia, 2=caveolae, 3=clathrin, 4=vesicle, 5=actin. **b**, An HSC3 apical plasma membrane on a Quantifoil R2/1 Au grid with an enlarged crop-out. **c,** An HSC3 basal plasma membrane on a Quantifoil R2/2 +2 nm carbon Au grid with an enlarged crop-out. **d,** The distribution of flat, dome, and sphere clathrin-coated structures for isolated basal plasma membranes from HSC3 cells grown on Quantifoil R2/2 +2 nm carbon Au grids. All images are tiled and stitched. The image in **a** is representative of N=4 grids, in **b**, N=4 grids, and in **c**, N=6 grids (basal, Quantifoil R2/2 +2 nm carbon Au). N=10 membranes from one grid were analyzed in **d**.

Figure S6.

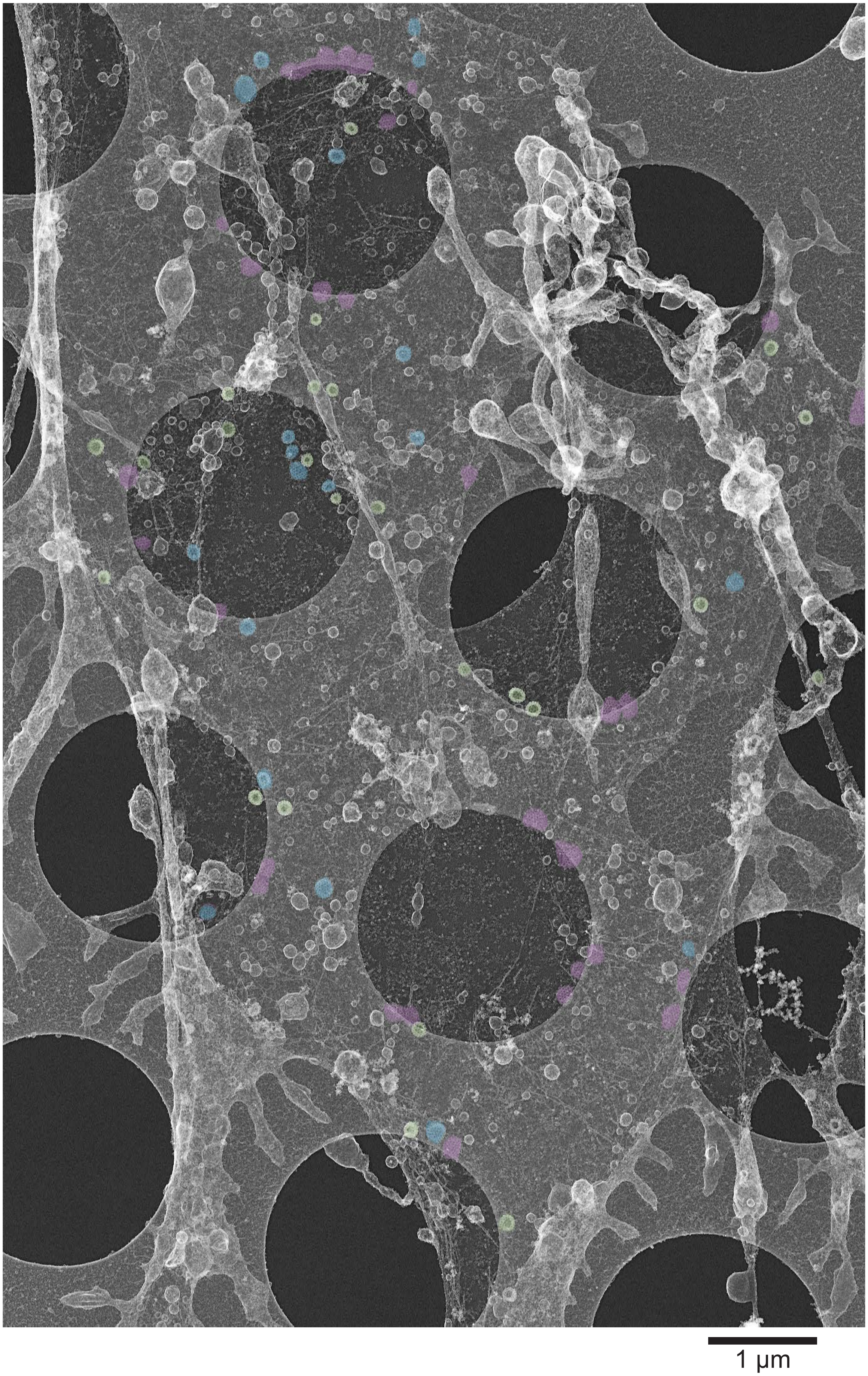

Figure S6. A large print of a 12.3 μm x 8 μm montage of an isolated basal plasma membrane from an HSC3 cell on an R2/1 Quantifoil grid. The three classes of clathrin structures are color labelled. Flat clathrin—lilac; dome—cyan; sphere—tea green.

Fig. S7

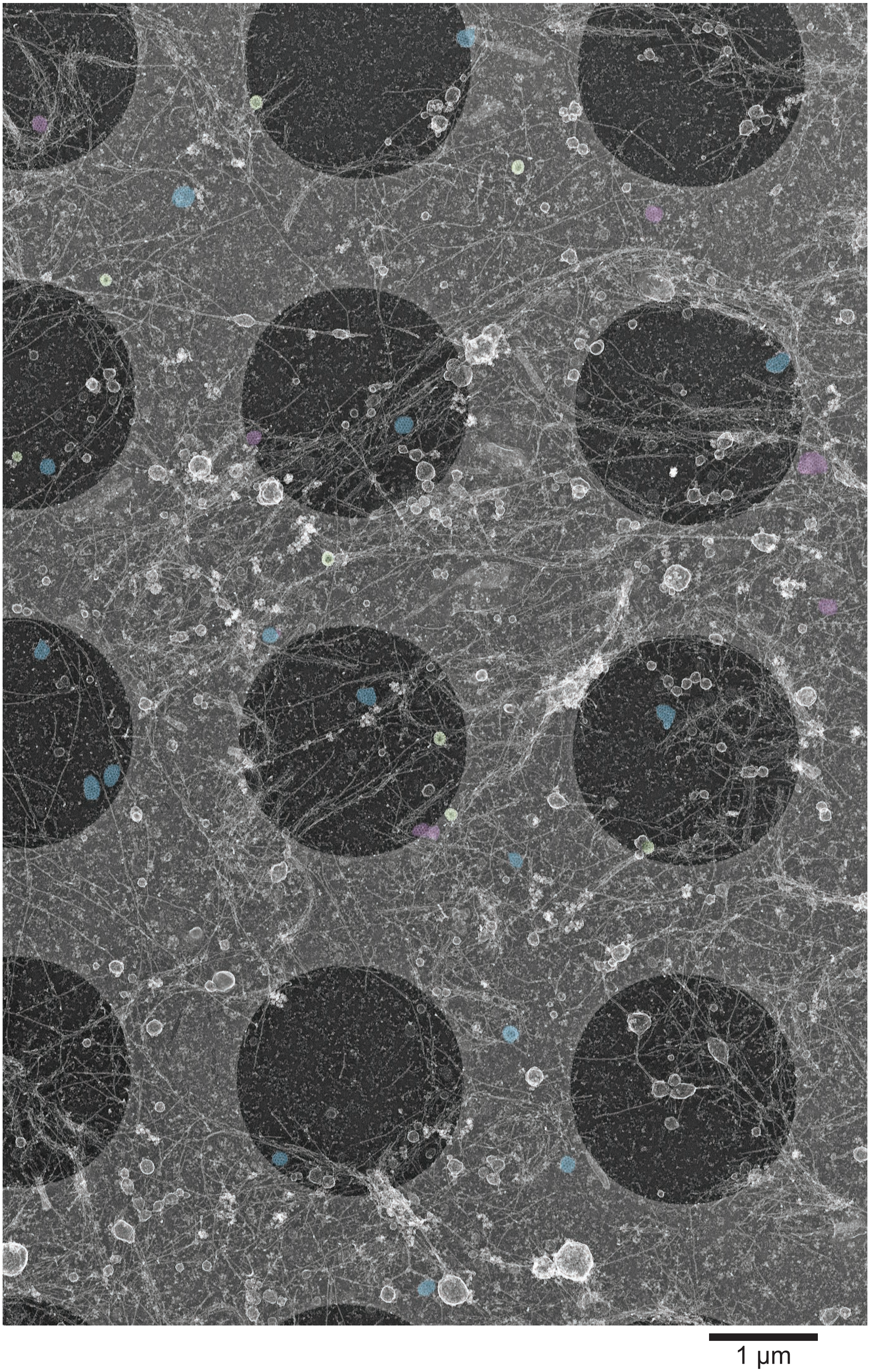

Figure S7. A large print of a 12.3 μm x 8 μm montage of an isolated apical plasma membrane from an HSC3 cell on an R2/1 Quantifoil grid. The three classes of clathrin structures are color labelled. Flat clathrin—lilac; dome—cyan; sphere—tea green.

Figure S8.

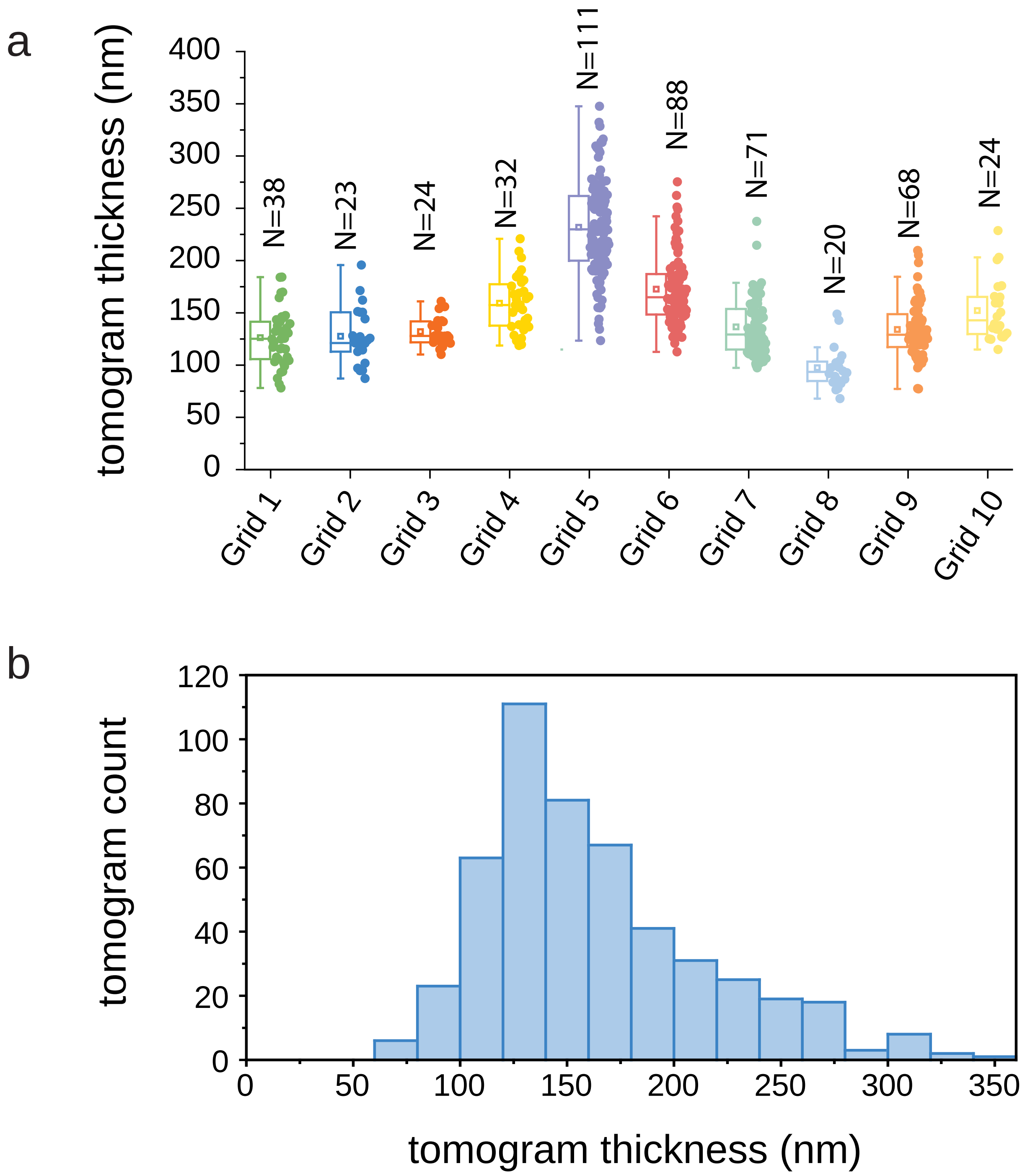

Figure S8. Tomogram thickness. a, The thickness of each cryo-tomogram used in this study grouped by grid. Grid numbers correlate with the numbers listed in Table 1 (grids 1-2, basal; grids 3-10, apical). Boxes indicate interquartile range with median (line), mean (square), and outlier range (whisker, coeff. 1.5). **b,** The same data as in a but combined into a single histogram.

Figure S9.

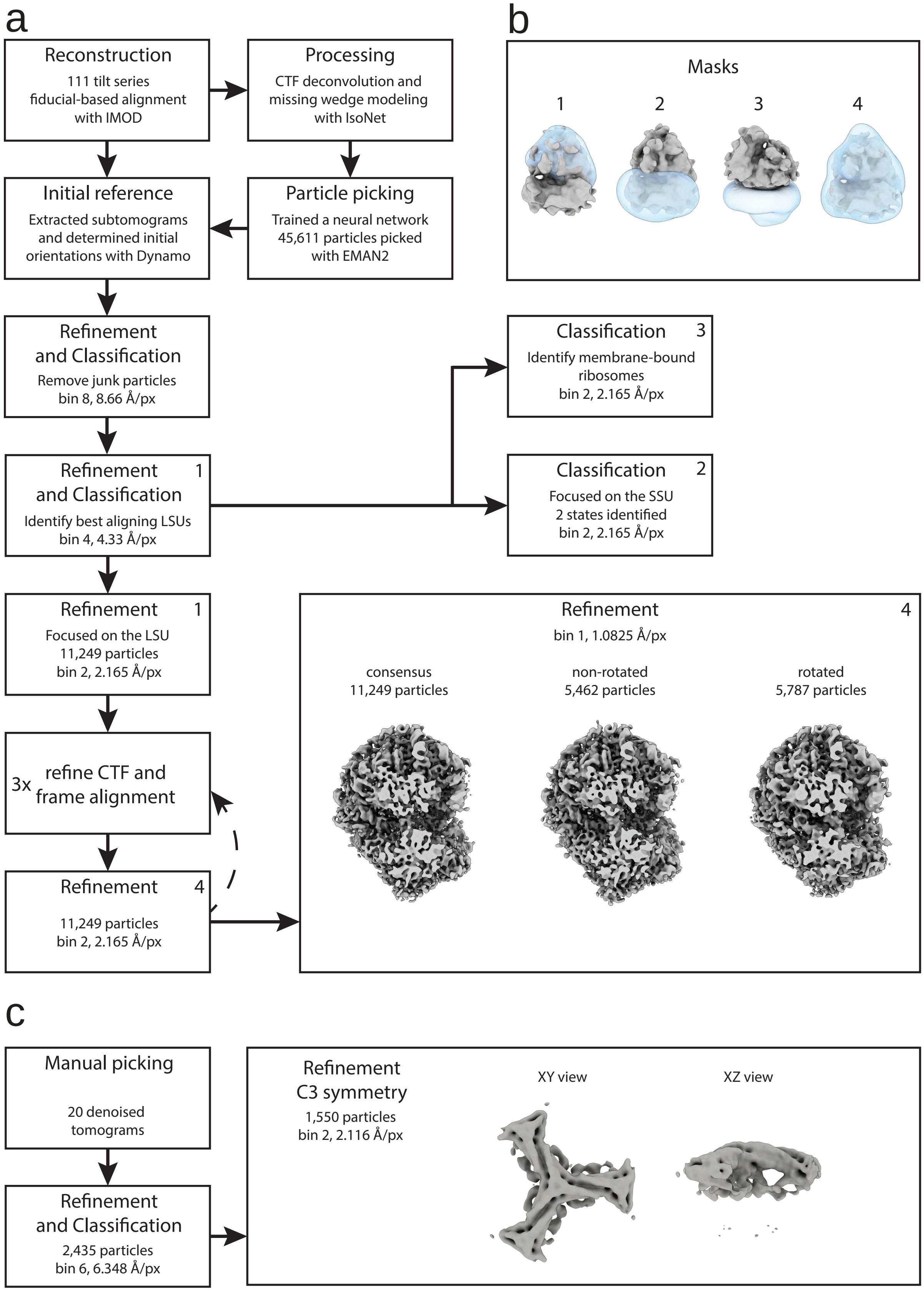

Figure S9. Visual representation of the subtomogram averaging workflow. a, Particles were subjected to multiple rounds of refinement and classification in RELION to remove junk particles and poorly aligning ribosomes. The rotated and non-rotated particle populations defined by the SSU-focused classification were used in subsequent refinements. **b,** Several masks were used in different processing steps focused on the ribosomal LSU (1), SSU (2), peptide exit tunnel and membrane region (3), and a full mask (4). A number in the upper right corner for a processing step indicates the mask applied. **c**, Subtomograms of manually picked clathrin were extracted, classified, and refined.

Figure S10.

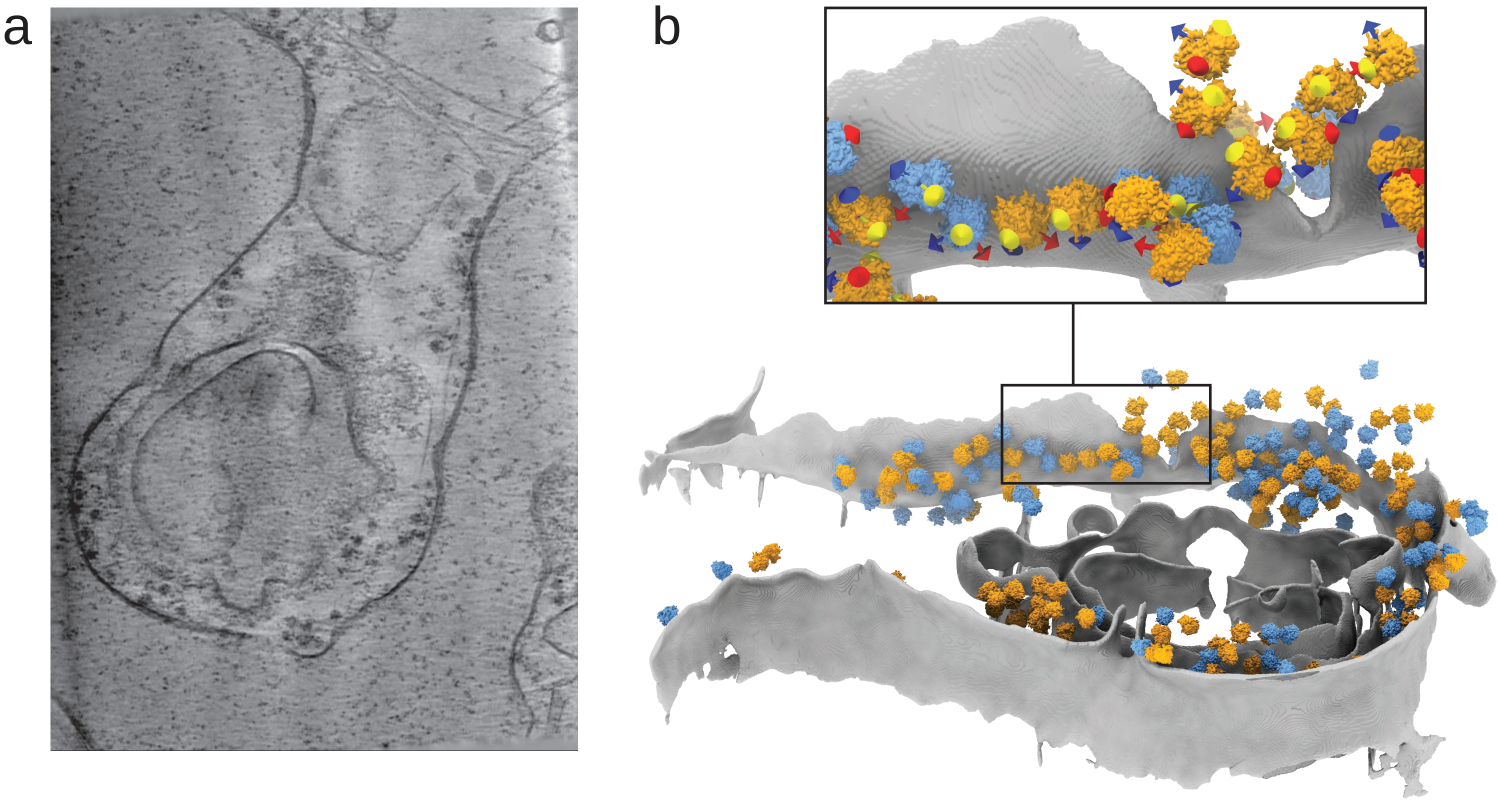

Figure S10. ER-bound polysome in unroofed membrane tomogram. **a**, Z-axis projection of 21 slices from a tomogram containing ER-bound ribosomes. **b**, Segmented ER membrane (gray) is shown with bound ribosomes. Averages from rotated (blue) and non-rotated (orange) classes are superimposed on particle positions from the membrane-bound particle set identified from classification. Similarly aligned orientation axes of neighboring ribosomes indicate a polysome (inset).

Figure S11.

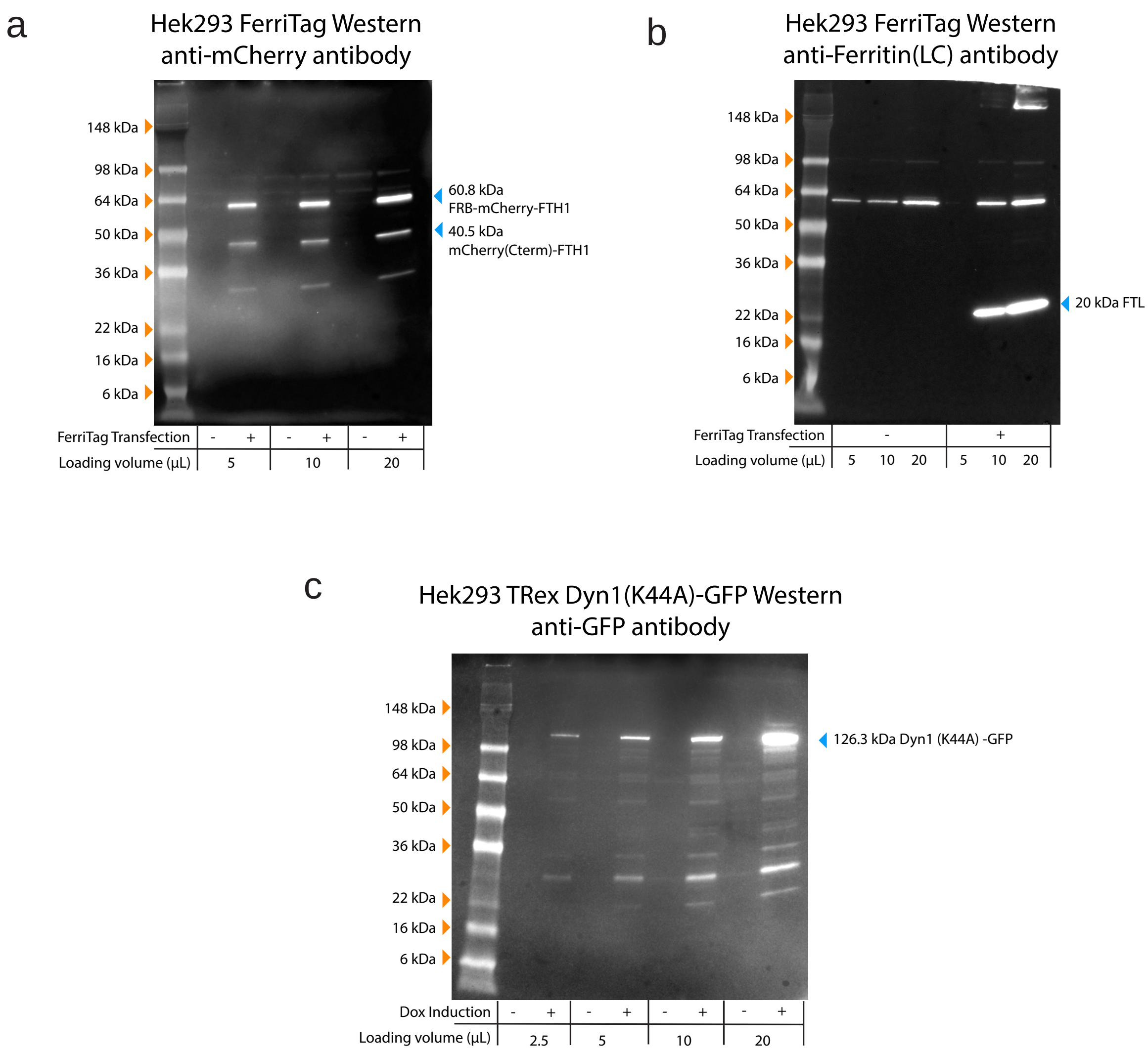

Figure S11. Western Blot Analysis of protein transfection. a) Western blot detection of mCherry (Novus Biologicals 1C51) in cell lysate from Hek293 cells not transfected or transfected with FerriTag confirms expression of FRB-mCherry-FTH1 (ferritin heavy chain). Fragmentation at the mCherry chromophore during boiling exhibits a predicable band that includes the c-terminus of mCherry and FTH1. b) Western blot detection of ferritin light chain (ProteinTech 10727-1-AP) in cell lysate from Hek293 cells not transfected or trans-fected with FerriTag confirms expression of FTL (ferritin light chain). c) Western blot detec-tion of GFP (Santa Cruz sc-9996) in cell lysate from the stable Hek293 Trex Dyn1 (K44A)-GFP cell line not induced or induced with doxycycline confirms expression of Dyn1 (K44A)-GFP.

Supplemental methods

Testing parameters for cell unroofing (Fig. S3)

Using the cell unroofing setup (Fig. S1), we evaluated how the pressure of the unroofing buffer and the distance between the sample and the syringe affect cell unroofing. HSC3-EGFR-GFP and MDA-MB231-CLC-GFP cells were seeded onto collagen- and fibronectin-coated coverslips, respectively, at 300k cell count and were incubated overnight at 37 ºC before undergoing cell unroofing. Seven pressures were tested: 0.1, 0.3, 0.5, 0.7, 0.8, 0.9 and 1 bar. Three coverslips were unroofed per pressure. Four distances were tested: 1, 2, 3, and 4 cm. Six coverslips were unroofed per distance. Unroofed samples were fixed with 2% PFA for 20 minutes, washed with 1X PBS, and stored in 1X PBS at 4 ºC before imaging.

Fluorescence microscopy (Fig. S3a)

Unroofed samples were imaged on a Nikon inverted fluorescence microscopy (Ti2 Eclipse, Nikon) using either 20x/numerical aperture (NA) 0.75 air or 20x/NA 0.45 air long working distance objectives. Epi-illumination was achieved with a solid-state white light source (SOLA Light Engine, Lumencor) and GFP signals were collected onto an Andor IXon Ultra 897 EMCCD. Montages of a large field of a coverslip were acquired using the Nikon NIS-Elements software.

Quantification (Fig. S3b-d)

The unroofed area was measured by taking advantage of the distinctive difference in fluorescence intensity between intact and unroofed cells. Montages were imported into FIJI and the radii and area of unroofed regions were measured using the fit ellipse function over unroofed areas. FIJI was also used for cropping and adjusting brightness/contrast. Area and radii measurements were recorded in Microsoft Excel and then imported into Prism (GraphPad) for data visualization.

Unroofing of SK-MEL-2 cells on grid (Fig. S3e-g)

SK-MEL-2-CLCa-GFP cells [1] were cultured in DMEM supplemented with 10% FBS. They were grown overnight onto Quantifoil R1.2/1.3 300 mesh gold grids and unroofed at a needle-sample distance of 1 cm with and buffer (stabilization buffer and 0.5% paraformaldehyde) pressurized at 0.7 bar. The unroofed grids were vitrified via plunge freezing using a Leica GP at 90% humidity and 3 seconds back blotting. The grids were then imaged under cryo-condition using a CryoCLEM Thunder Imager (Leica Microsystems).

Westerns (Fig. S11)

WT Hek293 cells were not transfected or transfected with the heavy chain and light chain components of FerriTag, *FRB-mCherry-FTH1* (addgene 100749) and *FTL* (addgene 100750) as described previously. Hek293 Trex Dyn1(K44A)-GFP expression was not induced or induced as described previously. Protein was extracted from cells using RIPA buffer supplemented with Halt protease inhibitor cocktail. Cells were incubated for 1 hour on ice. After centrifuging at 21300xg for 15 minutes, the supernatants were frozen and stored to use for westerns. Samples were run on Novex 10-20% TG Wedgewell 10W gels (Cat. XP10200BOX Invitrogen) using Tris/glycine/SDS buffer (Cat. 1610732 Bio-Rad) and a SeeBluePlus2 ladder (Cat. LC5925 Invitrogen) for reference. Varying volumes were used as described. Gels were run 20 minutes at 120 V, 90 mA, 12.5W and 50 minutes at 180 V, 90 mA, 12.5 W. The protein was transferred using mini Nitrocellulose iBlot Gel Transfer Stacks. Fig. S11a was WT Hek293 cells stained with anti-mCherry antibody (Novus Biologicals, 1C51, 1:1000; secondary, Jackson Immuno, 315-035-003, 1:10000). Fig. S11b was WT Hek293 cells stained with anti-FTL antibody (ProteinTech 10727-1-AP; secondary, Jackson Immuno 211-032-171, 1:10000). Fig. S11c was Hek293 Trex Dyn1(K44A)-GFP cells stained with anti-GFP antibody (Santa Cruz, sc-9996, 1:200; secondary, Jackson Immuno, 315-035-003, 1:10000). Blots were imaged after exposure to ECL Prime Luminol enhancer and peroxide solutions (Cytiva RPN2232V1, RPN2232V2).

References:

1. Serwas, D., et al., *Mechanistic insights into actin force generation during vesicle formation from cryo-electron tomography.* Dev Cell, 2022. **57**(9): p. 1132-1145.e5.
