# Supplementary material for: Cryo-electron tomography pipeline for plasma membranes
Source: Nat Commun. 2025 Jan 20;16:855. doi: 10.1038/s41467-025-56045-z (PMC11747107; doi:10.1038/s41467-025-56045-z)
Supplement: Supplementary file 2 — Description Of Additional Supplementary Files [file 41467_2025_56045_MOESM2_ESM.pdf]

Description of Additional supplementary files

Supplementary Movie 1. A bin 8 (voxel=0.86 nm) tomogram stack of the clathrin structure displayed in Fig. 6d. FerriTag is labelling the N-terminus of Hip1R. The hollow 12 nm spheres are FerriTag. The filaments surrounding the structure are actin (identified by ~7 nm diameter, 2.7 nm between subunits). The centralized clathrin pit is identified by engulfed membrane that is coated with a polyhedral coat, roughly 25 nm thick.

Supplementary Movie 2. A bin 8 (voxel=0.86 nm) tomogram stack of the clathrin structure displayed in Fig. 6e. FerriTag is labelling clathrin light chain. The hollow 12 nm spheres are FerriTag. The filaments surrounding the structure are actin (identified by ~7 nm diameter, 2.7 nm between subunits). There are two centralized clathrin vesicles identified by membrane vesicles coated with a polyhedral coat, roughly 25 nm thick.

Supplementary Movie 3. A bin 8 (voxel=0.86 nm) tomogram stack of the clathrin structure displayed in Fig. 6g. FerriTag is labelling the N-terminus of Hip1R. The hollow 12 nm spheres are FerriTag. The filaments surrounding the structure are actin (identified by ~7 nm diameter, 2.7 nm between subunits). The centralized clathrin pit is identified by engulfed membrane that is coated with a polyhedral coat, roughly 25 nm thick.

Supplementary Movie 4. A bin 8 (voxel=0.86 nm) tomogram stack of the clathrin structure displayed in Fig. 6h (left). FerriTag is labelling the Nterminus of Hip1R. The hollow 12 nm spheres are FerriTag. The filaments surrounding the structure are actin (identified by ~7 nm diameter, 2.7 nm between subunits). The centralized clathrin pit is identified by engulfed membrane that is coated with a polyhedral coat, roughly 25 nm thick. The arrow indicates the putative Hip1R density being featured in the main figure.

Supplementary Movie 5. A bin 8 (voxel=0.86 nm) tomogram stack of the clathrin structure displayed in Fig. 6h (right). FerriTag is labelling the Nterminus of Hip1R. The hollow 12 nm spheres are FerriTag. The filaments surrounding the structure are actin (identified by ~7 nm diameter, 2.7 nm between subunits). The centralized clathrin pit is identified by engulfed membrane that is coated with a polyhedral coat, roughly 25 nm thick. The arrow indicates the putative Hip1R density being featured in the main figure.

Supplementary Movie 6. A bin 8 (voxel=0.86 nm) tomogram stack of the clathrin structure that is not featured in a main figure. FerriTag is labelling the N-terminus of Hip1R. The hollow 12 nm spheres are FerriTag. The filaments surrounding the structure are actin (identified by ~7 nm diameter, 2.7 nm between subunits). The centralized clathrin pit is identified by engulfed membrane that is coated with a polyhedral coat, roughly 25 nm thick. The arrows indicate several putative Hip1R densities.
