# Supplementary material for: Cryo-electron tomography pipeline for plasma membranes
Source: Nat Commun. 2025 Jan 20;16:855. doi: 10.1038/s41467-025-56045-z (PMC11747107; doi:10.1038/s41467-025-56045-z)
Supplement: Supplementary file 9 — Reporting Summary [file 41467_2025_56045_MOESM9_ESM.pdf]

nature portfolio

Corresponding author(s): Kem A. Sochacki, Jenny E. Hinshaw, Justin W. Taraska

Last updated by author(s): Oct 10, 2024

Reporting Summary

Nature Portfolio wishes to improve the reproducibility of the work that we publish. This form provides structure for consistency and transparency in reporting. For further information on Nature Portfolio policies, see our Editorial Policies and the Editorial Policy Checklist.

Statistics

For all statistical analyses, confirm that the following items are present in the figure legend, table legend, main text, or Methods section.

n/a	Confirmed	
☐	☒	The exact sample size (*n*) for each experimental group/condition, given as a discrete number and unit of measurement
☐	☒	A statement on whether measurements were taken from distinct samples or whether the same sample was measured repeatedly
☐	☒	The statistical test(s) used AND whether they are one- or two-sided<br>*Only common tests should be described solely by name; describe more complex techniques in the Methods section.*
☒	☐	A description of all covariates tested
☒	☐	A description of any assumptions or corrections, such as tests of normality and adjustment for multiple comparisons
☐	☒	A full description of the statistical parameters including central tendency (e.g. means) or other basic estimates (e.g. regression coefficient) AND variation (e.g. standard deviation) or associated estimates of uncertainty (e.g. confidence intervals)
☐	☒	For null hypothesis testing, the test statistic (e.g. *F*, *t*, *r*) with confidence intervals, effect sizes, degrees of freedom and *P* value noted<br>*Give P values as exact values whenever suitable.*
☒	☐	For Bayesian analysis, information on the choice of priors and Markov chain Monte Carlo settings
☒	☐	For hierarchical and complex designs, identification of the appropriate level for tests and full reporting of outcomes
☒	☐	Estimates of effect sizes (e.g. Cohen's *d*, Pearson's *r*), indicating how they were calculated

Our web collection on statistics for biologists contains articles on many of the points above.

Software and code

Policy information about availability of computer code

Data collection	Data was collected using SerialEM, Nikon Elements.
Data analysis	ChimeraX 1.6 and 1.7, IMOD 4.12, RELION 4.0,RELION5.0, EMAN2 2.99, ImageJ 1.54f, Napari 0.4.18, Python 3, maskR-CNN 2.1, Matlab R2022b, dynamo 1.1.532, Isonet 0.2, https://github.com/dmichalak/sta-pipeline, https://github.com/KASochacki/clemposo.Code for clathrin segmentation in platinum replica data is available at https://github.com/andreasmarnold/PyCLEM (10.5281/zenodo.14391733, 2024).

For manuscripts utilizing custom algorithms or software that are central to the research but not yet described in published literature, software must be made available to editors and reviewers. We strongly encourage code deposition in a community repository (e.g. GitHub). See the Nature Portfolio guidelines for submitting code & software for further information.

Data

Policy information about availability of data

All manuscripts must include a data availability statement. This statement should provide the following information, where applicable:

- Accession codes, unique identifiers, or web links for publicly available datasets
- A description of any restrictions on data availability
- For clinical datasets or third party data, please ensure that the statement adheres to our policy

Platinum replica data are available on Figshare (https://nhlbi.figshare.com/; DOI: 10.25444/nhlbi.28012229). Binned tomograms and raw frames are available on

the Chan Zuckerberg Initiative CryoET Data Portal under accession code CZCDP-10306. Ribosome structures are being made available as EMD-44921, EMD-44909, and EMD-44922. The clathrin structure is being made available as EMD-46973.

Research involving human participants, their data, or biological material

Policy information about studies with human participants or human data. See also policy information about sex, gender (identity/presentation), and sexual orientation and race, ethnicity and racism.

Reporting on sex and gender	Gender of cell lines has been reported.HSC3 cells are from male origin and HEK293 cells are from fetal female origin (considered female due to lack of Y chromosome).
Reporting on race, ethnicity, or other socially relevant groupings	Ethnicity of cell lines has been reported. HSC3 cells are Japanese in origin HEK293 cells were acquired in the Netherlands but their ethnicity is unknown.
Population characteristics	NA
Recruitment	NA
Ethics oversight	NA

Note that full information on the approval of the study protocol must also be provided in the manuscript.

Field-specific reporting

Please select the one below that is the best fit for your research. If you are not sure, read the appropriate sections before making your selection.

☒ Life sciences ☐ Behavioural & social sciences ☐ Ecological, evolutionary & environmental sciences

For a reference copy of the document with all sections, see nature.com/documents/nr-reporting-summary-flat.pdf

Life sciences study design

All studies must disclose on these points even when the disclosure is negative.

Sample size	Sample sizes are all listed in the manuscript where applicable.
Data exclusions	After tilt series acquisition, data was processed to tomograms using a batch algorithm. At this stage, tomograms reconstructions that failed, had grid bar shadows in the tomogram, had no biological material, had ice contamination or sample damage were rejected. For STA, exclusion of ribosomes is clearly stated in the manuscript (ribosomes adhered to the underside of the carbon were manually removed). For FerriTag, tags at the edge of the tomograms and at the air water interfaces were not included in analysis as described in the manuscript.
Replication	The number of grids used for each Figure is listed in the manuscript, Table 1 also gives a clear explanation of what data were used. Each grid was replicated as shown in Table 1.
Randomization	NA
Blinding	NA

Reporting for specific materials, systems and methods

We require information from authors about some types of materials, experimental systems and methods used in many studies. Here, indicate whether each material, system or method listed is relevant to your study. If you are not sure if a list item applies to your research, read the appropriate section before selecting a response.

Materials & experimental systems

n/a	Involved in the study	
☐	☒	Antibodies
☐	☒	Eukaryotic cell lines
☒	☐	Palaeontology and archaeology
☒	☐	Animals and other organisms
☒	☐	Clinical data
☒	☐	Dual use research of concern
☒	☐	Plants

Methods

n/a	Involved in the study	
☒	☐	ChIP-seq
☒	☐	Flow cytometry
☒	☐	MRI-based neuroimaging

Antibodies

Antibodies used	Novus Biologicals, 1C51; Jackson Immuno, 315-035-003; ProteinTech 10727-1-AP; Jackson Immuno 211-032-171; Santa Cruz, sc-9996
Validation	antibodies were only used for westerns to confirm expression of plasmids. The bands ran at the expected MW.

Eukaryotic cell lines

Policy information about cell lines and Sex and Gender in Research

Cell line source(s)	Hek-293 and MDA-MB-231 was obtained through ATCC, Trex293 was obtained through Invitrogen, HSC3 EGFR-GFP was obtained from Dr. Alexander Sorkin's lab.
Authentication	Hek293, HSC3 cells, and MDA-MB231-CLC-GFP were authenticated by ATCC.
Mycoplasma contamination	Hek293, HSC3 cells, and MDA-MB231-CLC-GFP tested negative for mycoplasma.
Commonly misidentified lines (See ICLAC register)	No commonly misidentified cell lines were used in this study to our knowledge.

Plants

Seed stocks	NA
Novel plant genotypes	NA
Authentication	NA
